# Dietary and Metabolic Approaches for Treating Autism Spectrum Disorders, Affective Disorders and Cognitive Impairment Comorbid with Epilepsy: A Review of Clinical and Preclinical Evidence

**DOI:** 10.3390/nu16040553

**Published:** 2024-02-17

**Authors:** Shruthi H. Iyer, Mary Y. Yeh, Lauren Netzel, Molly G. Lindsey, McKenzie Wallace, Kristina A. Simeone, Timothy A. Simeone

**Affiliations:** Department of Pharmacology & Neuroscience, Creighton University School of Medicine, Omaha, NE 68178, USA; shruthiiyer@creighton.edu (S.H.I.); kristinasimeone@creighton.edu (K.A.S.)

**Keywords:** autism, affective disorder, cognitive impairment, ketogenic diet, MCT, low glycemic index, calorie restriction, PUFA, ketone, triheptanoin

## Abstract

Epilepsy often occurs with other neurological disorders, such as autism, affective disorders, and cognitive impairment. Research indicates that many neurological disorders share a common pathophysiology of dysfunctional energy metabolism, neuroinflammation, oxidative stress, and gut dysbiosis. The past decade has witnessed a growing interest in the use of metabolic therapies for these disorders with or without the context of epilepsy. Over one hundred years ago, the high-fat, low-carbohydrate ketogenic diet (KD) was formulated as a treatment for epilepsy. For those who cannot tolerate the KD, other diets have been developed to provide similar seizure control, presumably through similar mechanisms. These include, but are not limited to, the medium-chain triglyceride diet, low glycemic index diet, and calorie restriction. In addition, dietary supplementation with ketone bodies, polyunsaturated fatty acids, or triheptanoin may also be beneficial. The proposed mechanisms through which these diets and supplements work to reduce neuronal hyperexcitability involve normalization of aberrant energy metabolism, dampening of inflammation, promotion of endogenous antioxidants, and reduction of gut dysbiosis. This raises the possibility that these dietary and metabolic therapies may not only exert anti-seizure effects, but also reduce comorbid disorders in people with epilepsy. Here, we explore this possibility and review the clinical and preclinical evidence where available.

## 1. Introduction

A comorbidity is defined as the prevalence of one or more additional symptoms, syndromes, or an entire disease that exists in addition to a primary disease or disorder. According to meta-analyses of studies involving close to 2 million people, it is estimated that between 26 and 84% of people with epilepsy have at least one comorbidity [1,2,3,4,5]. This range reflects the contribution of comorbid conditions to our evolving conceptualization of epilepsy as a spectrum disorder. Even though comorbidities have been estimated to be up to eight times higher in epilepsy, many neurology clinics lack appropriate screening; thus, this is likely an underestimate. 

In general, the pharmacological treatment of epilepsy and its comorbidities is approached independently, with priority given to achieving seizure freedom. It has been observed that some antiseizure medications may worsen comorbidity severity. Thus, while drug-mediated seizure control is important in the symptomatic management of epilepsy, there is a need for treatments with a broader range of mechanistic action that can also influence comorbid conditions. 

In the past two decades, the investigation of novel therapeutic approaches suggests that targeting the metabolic machinery effectively reestablishes neuronal network homeostasis, normalizes aberrant energy metabolism, dampens inflammation, and promotes endogenous antioxidants [6,7,8,9,10,11]. Metabolic therapies may also provide etiological relief by modulating essential microbiota in the gut, as gut dysbiosis, or dysregulation in gut microbiota and their metabolites, has been implicated in the pathophysiology of intractable epilepsy and neurological comorbidities [12,13,14,15,16,17,18,19,20,21,22]. Several preclinical and clinical studies have found that metabolic therapies can attenuate seizures and, in some instances, abolish epilepsy [23,24]. Since many of the comorbidities in epilepsy share a common neurobiological pathology, it is reasonable to hypothesize that the metabolic therapies that alter the pathophysiology in epilepsy may also improve some neurological conditions comorbid with epilepsy. Here, we review the type and prevalence of a variety of comorbidities that are commonly experienced by patients with epilepsy, including autism, cognitive impairment, and affective disorders. We provide a summary of the known impacts of metabolic therapies on these comorbidities in preclinical animal models and clinical subjects where available. The overall aim is to review the influence of metabolic therapies on these specific comorbidities, regardless of the impact on seizures, in order to assess whether their inclusion in the armamentarium of wholistic epilepsy management warrants further consideration. We focus on dietary manipulations, namely the ketogenic diet, medium-chain triglyceride diet, low glycemic index diet, and calorie restriction, all of which have been clinically proven to reduce seizures in epilepsy. Dietary manipulation can be too difficult for some individuals; therefore, where evidence exists, we also review the alternative supplements, such as ketone bodies, polyunsaturated fatty acids, and triheptanoin, which may prove more practical in certain circumstances. We searched the preclinical and clinical literature using a combination of keywords for each metabolic therapy (including ketogenic, diet, medium-chain triglyceride, low glycemic index, caloric restriction, polyunsaturated fatty acids, ketone bodies, beta hydroxybutyrate, and triheptanoin) and each comorbidity (autism, cognition, learning, memory, affective disorder, anxiety, and depression) to ensure that all relevant literature was included. We utilized the PubMed and Scopus search engines and limited inclusion to English-language articles published until December 2023 involving human case reports, clinical trials and meta-analyses, and animal research studies. Overall, our narrative review provides intriguing evidence suggesting that dietary and metabolic therapies may improve autism, affective disorders, and cognitive impairment comorbid with epilepsy; however, it also highlights that there is a significant need for further preclinical and clinical studies for each potential therapy (Table 1).

## 2. Autism Spectrum Disorder

Autism is roughly 20% more frequent in patients with epilepsy (7–46%) when compared to the general population (0.7–1%) and the figures are projected to increase as the inclusion criteria for Autism Spectrum Disorder (ASD) broaden [25,26,27,28]. The relationship between autism and epilepsy is poorly understood and current treatments approach the two disorders independently. Common pharmacological treatments for epilepsy, such as levetiracetam and topiramate, can have negative impacts on the core symptoms of ASD, including cognition, mood, and behavior [28]. Though some of the behavioral and emotional symptoms associated with ASD can be treated with psychotropic medications—which may have substantial adverse effects—there are currently no efficacious pharmacological treatments for the core symptoms involving social deficits and restricted or repetitive behaviors or interests. 

Metabolism and energy processes play an important role in both neuronal network development and maintenance. Dysregulation of metabolism has been implicated in the ASD pathophysiology. In addition, the consumption of sugar has been shown to aggravate symptoms in ASD [29]. Promising data suggest a place for metabolic therapies in the armamentarium for ASD comorbid with epilepsy.

### 2.1. Ketogenic Diet (KD)

Clinically prescribed ketogenic diets are high-fat, low-carbohydrate/protein diets that range from 4:1 to 2:1 fats:carbohydrate/protein depending on the therapeutic needs and tolerability of the patient. Dietary fats in a classic KD are mostly long-chain triglyceride fats and provide 60–80% of energy. One of the hallmarks of the KD is an increase in blood ketone bodies (beta-hydroxybutyrate, acetoacetate, and acetone) and polyunsaturated fatty acids, and a decrease in blood glucose. These biochemical products of a KD are thought to play a role in the mechanism of action to reduce seizures and are under intense study [30]. Though systematic clinical studies concerning the use of KD in autism comorbid with epilepsy are sparse, preliminary clinical evidence is promising. A key pathophysiology manifested in autism is brain glucose dysregulation. This presents in the form of both the hyper- and hypometabolism of glucose in specific brain regions, which can translate into specific behavior phenotypes [31]. A recent case study found that one month after KD treatment, attention span, communication skills, and emotional reactions were improved and hyperactivity and aggressive behavior was decreased in a six-year-old patient with autism and subclinical epileptic discharges [31]. The patient also showed an increase in full-scale IQ, as measured by the Wechsler Intelligence Scale for Children—Revised (WISC-R), with a significant improvement after a short period. In several brain regions, brain glucose hypometabolism measured via positron emission tomography (PET) was normalized to an extent after 12 months of KD consumption. Other case reports also demonstrate the similar amelioration of ASD symptoms during a KD [32,33]. 

Preclinical studies have demonstrated that a KD improves sociability and social communication, as well as ameliorating self-directed repetitive behavior in various murine models of ASD, including BTBR mice, the maternal immune activation (MIA) mouse model of ASD, and the valproic acid mouse model [34,35,36]. In a mouse model of ASD with epilepsy, EL mice, the KD improved performance on standard preclinical behavioral tests developed as correlates of ASD core symptoms, including the three-chamber sociability test, social novelty test, and self-grooming test [36].

Mitochondrial dysfunction has been identified as one of the possible mechanisms playing a role in epilepsy and ASD. A preclinical study found mitochondrial dysfunction in the valproic acid ASD mouse model [37]. KD treatment improved some aspects of this dysfunction [20]. Similarly, KD treatment for two weeks improved several mitochondrial endpoints in BTBR mice, including increasing the basal oxygen consumption rate and ATP production and stabilizing the abnormally increased phosphorylation of AMPK [38]. In addition, mitochondrial fragmentation has been observed in ASD and found in BTBR mice. KD treatment restored the mitochondrial morphology in these mice, by reducing the phosphorylation of DRP1S616, the fission protein involved in mitochondrial fragmentation [39].

### 2.2. Medium-Chain Triglyceride (MCT) Diet

MCT is an alternative type of ketogenic diet that relies on the premise that medium-chain fatty acids such as octanoic acid (also known as caprylic acid) and decanoic acid (also known as capric acid) are metabolized into ketone bodies more efficiently and allow a more palatable diet with higher consumption of carbohydrates [40]. During an MCT diet, 30–60 percent of the calories are obtained from MCT oil or emulsion and 30 percent from long-chain dietary fats. Though MCT diets are effective in treating epilepsy [41], there is a lack of clinical and preclinical studies available on autism and epilepsy. Interestingly, one case report followed a child with autism and epilepsy placed on a gluten-free, casein-free MCT diet for several years and reported a remarkable reduction in the Childhood Autism Rating Scale score from 49 to 17, representing a change from severe autism to non-autistic and seizure freedom [33]. 

### 2.3. Low Glycemic Index (LGI) Diet

Although sugar may aggravate symptoms in ASD, no published clinical studies using LGI are available as of the time of writing. There are also limited preclinical studies. However, one promising study provides substantial evidence for the use of the LGI diet to treat symptoms of ASD in the BTBR mouse model of autism [42]. The study found that the LGI diet reduced the characteristic behavioral markers of ASD on spatial tasks, repetitive self-grooming, and deficits in social interaction. It was also found that many indicators of inflammation and oxidative stress were upregulated in the BTBR model of ASD, including C-reactive protein (CRP), advanced glycation end products, and electrophilic dicarbonyl methylglyoxal. The levels of these indicators were reduced in BTBR mice treated with an LGI diet [42]. These data indicate that a low GI diet may attenuate the ASD behavioral phenotype by reducing inflammation and oxidative stress.

### 2.4. Calorie Restriction Diet

No published clinical studies using calorie restriction to treat autism comorbid with epilepsy are available as of the time of writing; however, a preclinical study of *Mecp2^308/y^* mice, a model of Rett syndrome with ASD and epilepsy, found that calorie restriction reduced the elevated anxiety phenotype of these mice [43].

### 2.5. Ketone Body Supplementation

Ketone bodies are significantly elevated during ketogenic diet treatment; thus, studies are beginning to determine whether pharmacological supplementation with ketone bodies can exert similar effects. At present, studies are limited to preclinical models. Angelman Syndrome is characterized by intellectual disability, autism, and epilepsy. In a mouse model of Angelman Syndrome, ketone ester supplementation (with R,S-1,3-butanediol acetoacetate diester) improved motor coordination, object recognition, fear conditioning, associative memory, and hippocampal LTP, as well as reducing seizures [44]. Similar to the KD, treatment with another ketone body, β-hydroxybutyrate, improved mitochondrial function, suggesting an important mechanism of action for the treatment of these disorders [6,30,38].

### 2.6. Polyunsaturated Fatty Acid (PUFA) Supplementation

Clinical studies indicate decreased blood levels of omega-3 and omega-6 PUFAs in ASD, attributed to disturbances in the fatty acid metabolism combined with poor dietary intake of these essential fatty acids. A meta-analysis study reported that ASD patients had lower levels of omega-3 PUFAs like docosahexaenoic acid (DHA), arachidonic acid (ARA), and eicosapentaenoic acid (EPA) [45]. Clinical data on PUFA supplementation are scarce; however, omega-3 fatty acid supplementation seems to improve the ASD core symptoms, like social interaction, repetitive and restrictive behaviors, and hyperactivity [45,46,47,48,49]. 

Omega-3 PUFAs are thought to be beneficial in many diseases due to their anti-inflammatory properties. Currently, reports of PUFA use in preclinical models of autism comorbid with epilepsy are lacking. However, their effectiveness has been investigated in models of autism alone. Prenatal exposure to lipopolysaccharide, the viral mimetic polyinosinic:polycytidylic, or valproic acid resulted in neuroinflammation and autism-like changes in behavior in offspring [50,51,52,53]. Dietary supplementation with EPA and/or DHA reversed these behaviors and reduced markers of neuroinflammation, such as tumor necrosis factor alpha (TNFa) and interleukin-6 (IL-6). Importantly, these inflammatory cytokines are also involved in excitotoxicity and increased susceptibility to seizures. Omrani and colleagues [54] found that EPA and DHA supplementation in patients with refractory epilepsy experienced decreased seizures as well as TNF-alpha and IL-6 concentrations, suggesting a mechanistic commonality between ASD and epilepsy that can be targeted using PUFA supplementation [54].

### 2.7. Triheptanoin Supplementation

Triheptanoin is a triglyceride of 7-carbon heptanoic acid that can replenish TCA cycle intermediates in a process called anaplerosis. It is used to treat pyruvate carboxylase deficiency and carnitine palmitoyltransferase II deficiency and is under investigation for the treatment of epilepsy, but there are no published clinical studies using triheptanoin to treat autism comorbid with epilepsy available to date. A preclinical study has evaluated triheptanoin in the MeCP2 knockout mouse model of Rett syndrome (RTT), which is part of ASD. Triheptanoin increased longevity; improved sociability, rotarod performance, and basal oxidative metabolism; decreased the abnormally high serum leptin and insulin levels; and improved the mitochondrial morphology and content and overall metabolism in these Mecp2 KO mice [7].

## 3. Affective Disorders

Epilepsy has been estimated to have comorbidity rates of 9–37% with depression and 11–25% with anxiety [55]. While there are many drugs that treat depression, anxiety, and epilepsy individually, there are few treatment options that have shown efficacy for both affective disorders and epilepsy. Clinical reports are sparse; therefore, we primarily review the relevant preclinical data regarding metabolic treatments for depression and anxiety with or without epilepsy.

### 3.1. Ketogenic Diet (KD)

The KD’s effects on affective disorders in people with epilepsy have not been exhaustively studied; however, a randomized clinical trial of children and adolescents with pharmacoresistant epilepsy, but not a clinically diagnosed affective disorder per se, did find that patients on a KD had decreased depressive and anxiety symptoms independent of seizure control [56]. Moreover, small trials and case reports suggest that a KD can ameliorate depressive symptoms in major depression disorder and bipolar disorder [57,58,59]. 

Preclinical evidence is equally scarce. Murphy et al. evaluated the antidepressant activity of the KD using the forced swim test on mice [60]. The study found that the KD group spent less time immobile when compared to animals on a control diet. The reduction in learned helplessness was similar to that in mice treated with antidepressant medications. Another study demonstrated that prenatal exposure to KD (via the administration of KD to pregnant CD1 mouse dams) altered depression-like behavior in adult offspring. The study found that adult mice exposed to prenatal KD had reduced learned helplessness and increased physical activity during the exercise wheel test. Anxiety behaviors were also decreased as the KD-treated mice were less anxious about visiting the center of the arena compared with the standard diet controls [61].

### 3.2. Medium-Chain Triglyceride (MCT) Diet

No published clinical studies using MCT for either an affective disorder comorbid with epilepsy or an affective disorder alone are available as of the time of writing. In a preclinical study using male Wistar rats, MCT was found to reduce anxiety-like behavior in the light-dark test and increase social competitiveness in the social dominance test. There was no difference between the MCT group and the control group for the forced swim test or the social exploration test [62]. A recent study found that the acute administration of a high dose of decanoic acid increased depression/anxiety in mice during the forced swim test [63]. In contrast, a seven-day treatment with virgin coconut oil, high in MCT, reduced immobility in the forced swim test and was associated with reduced serum cortisol and an increase in brain antioxidants [64].

### 3.3. Low Glycemic Index (LGI) Diet

A recent retrospective study has highlighted the importance of proper glycemic control in psychiatric health [65]. Kwon et al. examined a decade of health insurance-related screenings of over 150,000 people and found that high glycemic variability and persistent hyperglycemia were associated with the increased incidence of depression and anxiety disorders [65]. However, there are currently no published clinical or preclinical studies using the LGI diet for either an affective disorder comorbid with epilepsy or an affective disorder alone.

### 3.4. Caloric Restriction

The effect of calorie restriction on depression is of interest in both preclinical and clinical research. However, to date, only a limited number of studies have been performed, and none in animals or humans with both an affective disorder and epilepsy. A small clinical study reported that intermittent fasting in a group of 16 healthy geriatric male adults decreased tension, anger, confusion, and mood disturbances, and they had increased vigor [66]. These data support the notion that caloric restriction can have positive effects on affect and behavior. Moreover, there was no change in mean depression scores as measured on the Beck Depression Inventory and the Geriatric Depression Scale, measured throughout the course of the study, indicating that a 3-month calorie-restrictive diet by itself does not induce depression in healthy patients. A more recent meta-analysis of randomized and nonrandomized clinical trials examined the effect of intermittent fasting on affective disorders and mood. It was concluded that intermittent fasting improved depression scores, but anxiety and mood were not modified [67].

Preclinical studies in rodent models of depression consistently find that calorie restriction or intermittent fasting exerts antidepressant-like effects [68,69,70,71]. Evidence suggests that increased antioxidant and anti-inflammatory mediators, mitochondria biogenesis, decreased serotonin, and increased ATP, orexin, and ghrelin may be involved in the mechanism of action of calorie restriction [68,69,70,71,72]. In one particularly interesting study, acute fasting for nine hours reduced depressive behavior in mice in the forced swim test [70]. Pretreatment with the antidepressant imipramine, a serotonin and norepinephrine reuptake inhibitor, had similar effects. Combined fasting and imipramine pre-treatment had additive effects, leading to a further reduction in learned helplessness. These effects were reversed by the administration of DOI, a 5-HT2A/2C receptor agonist, indicating that fasting and imipramine may exert their antidepressant action via the modulation of the serotonergic 5HT-2A receptors [55]. Intriguingly, preclinical studies in epilepsy have indicated a pro-convulsive effect of the 5HT2A receptors [73]. Agonism at this receptor decreased the latency to generalized convulsions in a feline model of hippocampal kindled seizures and facilitated amygdala kindling in rats, while antagonism reduced the number of and latency to seizures in various preclinical animal models of epilepsy [74,75,76]. Together, these studies suggest the possibility that serotonin may be a common pathology shared by epilepsy and depression that can be modulated via caloric restriction to treat both disorders. 

### 3.5. Ketone Body Supplementation

No published clinical studies using ketone bodies for either an affective disorder comorbid with epilepsy or an affective disorder alone are available as of the time of writing. Limited preclinical studies have explored the use of ketone body or ketone ester treatment on rodent models of depression and anxiety. The few studies available report reduced depression and anxiety in the forced swim test, tail suspension test, elevated plus maze, and open field test in normal rodents and rodents exposed to chronic unpredictable mild stress [9,77,78,79]. The chronic focal infusion of beta-hydroxybutyrate into the prefrontal cortex had similar effects [80]. Ketone bodies’ effects on depression and anxiety were associated with decreases in inflammatory cytokines and reduced corticosterone [9,78,79,80]. 

### 3.6. Polyunsaturated Fatty Acid (PUFA) Supplementation

Studies have found that patients with depression have lower peripheral levels of omega-3 polyunsaturated fatty acids (PUFAs) [81,82,83]. Increasing evidence supports the potential therapeutic use of long-chain fatty acids in those with depression and anxiety. In a meta-analysis of randomized controlled clinical trials of males and females diagnosed with major depressive disorder or depressive symptoms, it was concluded that treatment with omega-3 PUFAs was effective in decreasing symptoms of depression [84]. A more recent meta-analysis found that omega-3 PUFA supplementation could be considered as an effective adjuvant for the treatment of depression [85]. 

Preclinical studies indicate that the supplementation of long-chain PUFAs has antidepressant/anxiolytic effects in the forced swim test, tail suspension, elevated plus maze, or open field test [86,87,88]. PUFAs also exert nutrigenetic effects by activating PPARgamma (peroxisome proliferator-activated receptor gamma), which is anti-inflammatory against cytokines like TNF-alpha and IL-1 β; the direct administration of these cytokines is sufficient to produce the complete spectrum of depressive disorder [10,11,89,90]. These inflammatory cytokines are incidentally increased in clinical and preclinical cases of temporal lobe epilepsies, and status epilepticus and PPARgamma activation has been shown to reduce seizures preclinically [91,92,93]. Thus, PUFA supplementation may target comorbid depression and epilepsy by targeting the common inflammatory pathways involved in both disorders.

### 3.7. Triheptanoin

No published clinical or preclinical studies using triheptanoin as a treatment for either an affective disorder comorbid with epilepsy or an affective disorder alone are available to date.

## 4. Cognitive Impairment

Patients with epilepsy of all ages often exhibit comorbid cognitive impairments [94,95]. The prevalence of comorbid cognitive dysfunction ranges from 15 to 40% and it can involve various cognitive domains, such as visual memory, attention, executive function, visuospatial skills, verbal memory, and language [96,97]. Currently, there are no pharmacological therapies for cognitive impairment comorbid with epilepsy. However, preclinical experiments have demonstrated that various metabolic therapies can significantly impact, and generally improve, cognition in animal models of epilepsy. Metabolic therapies have also been examined in other disorders with cognitive impairment, such as Alzheimer’s Disease (AD), autism, affective disorders, and ageing; therefore, we have also included these studies where appropriate. 

### 4.1. Ketogenic Diet (KD)

There have been two randomized controlled clinical trials focused on the KD’s effects on cognition in adult refractory epilepsy and AD; both found modest KD-mediated improvements [56,98]. A third randomized controlled clinical trial only enrolled healthy individuals and demonstrated that a KD had no effect on general cognitive performance [99]. A series of uncontrolled trials also found cognitive improvements during KD treatment for obesity, HIV-associated neurocognitive impairment, and Dravet Syndrome (DS) [100,101,102,103]; however, no change or worsening was reported for patients with Glut1-DS or pediatric refractory epilepsy [104,105,106]. In contrast, three retrospective studies reported improvements in patients with Glut1-DS and pediatric refractory epilepsy [107,108,109]. Overall, the clinical evidence suggests that a KD will have a positive impact on cognitive performance in various neurological disorders.

Preclinically, the KD has neuroprotective effects in both AD and epilepsy through inducing ketosis, reducing blood sugar concentrations and neuroinflammatory cytokines, increasing fatty acid oxidation and mitochondrial respiration, and reducing oxidative stress and ROS generation [6,8,30,110,111,112,113,114]. KD treatment improved spatial memory in the *Kcna1*-null mouse model of epilepsy, PTZ-kindled rats, a model of hypobaric hypoxia-induced memory impairment, and multiple sclerosis and even improved the performance of healthy animals [6,115,116,117,118,119,120]. In contrast, there are also reports indicating no effect of the KD in the APP + PS1 and Tg4510 mouse models of AD and healthy rodents, and it even worsened cognitive performance in models of status epilepticus-induced epilepsy [121,122,123,124]. 

### 4.2. Medium-Chain Triglyceride (MCT) Diet

An MCT diet had no effect on cognitive measures in a randomized controlled clinical trial in multiple sclerosis patients [125] but did improve the performance of elderly adults and AD patients in three other randomized controlled clinical trials [126,127,128]. An uncontrolled clinical trial also found that MCT improved cognition in AD patients [129]. In general, an MCT diet improves cognitive impairment in animal studies; however, the effects of specific MCT diets are still unclear. This is illustrated by the finding that an MCT based on the 10-carbon decanoic acid improved novel object recognition in healthy rats, whereas an 8-carbon octanoic acid MCT had no effect [130]. However, an MCT8 diet improved spatial learning and reversal learning in healthy aged beagles [131]. Furthermore, a rigorous prospective, randomized, double-blinded, controlled cross-over trial in canines with epilepsy used an MCT based on rapeseed oil containing 50–65% octanoic acid and 30–50% decanoic acid and reported a significant reduction in seizures and improved cognition in the dogs during MCT treatment [132].

### 4.3. Low Glycemic Index (LGI) Diet

No published clinical or preclinical studies using the LGI to treat cognitive impairment comorbid with epilepsy are available as of the time of writing.

### 4.4. Calorie Restriction

Although calorie restriction and intermittent fasting have proven health benefits, there are few studies determining the impact on cognition with or without epilepsy. Two clinical studies tested the verbal memory scores of elderly individuals on a calorie-restricted diet over 3 months and found significant improvements in memory performance [133,134]. A large, multicenter, randomized clinical trial in non-obese healthy volunteers titled the Comprehensive Assessment of Long-Term Effects of Reducing Intake of Energy (CALERIE) demonstrated significant improvements in spatial working memory over two years of calorie restriction; however, a secondary analysis of CALERIE normalized the spatial working memory outcomes to the dietary quality and failed to demonstrate a significant difference between calorie restriction and the control treatment [135]. Thus, further trials are required to discern the effects on health and disease.

Preclinical studies have provided more consistent results regarding calorie restriction and cognition. Calorie restriction enhanced spatial learning in the Morris water maze in healthy mice and in the radial arm water maze task in healthy old mice [136,137,138]. Young and old rats on CR prior to focal ischemia were partially protected against the spatial memory deficits in the Morris water maze that appear following stroke induction [139]. The improvements due to calorie restriction may be mediated by the inhibition of the mammalian target of rapamycin (mTOR) signaling pathway [138]. Incidentally, hyperactive mTOR signaling is associated with epilepsy, and inhibitors of mTOR result in seizure reductions in several animal models of epilepsy and patients with epilepsy [140,141]. The inhibition of mTOR pathways leads to a metabolic change from higher glucose utilization to more fatty acid utilization, increased ketone bodies, and reduced oxidative stress and neuroinflammation. These mechanisms have been implicated in improving cognition and reducing the rate of epileptogenesis, as well as the comorbid cognitive deficits seen in epileptic models and clinical cases [141,142,143,144,145,146]. Another mechanism may be through increased levels of metalloproteinase ADAM10. Wang et al. found that in the Tg2576 mice model of AD, calorie restriction prevented beta-amyloid plaque development in the cortical and hippocampal regions of the brain via increased levels of ADAM10 and anti-amyloidogenic alpha secretase activity [147]. Multiple studies have found that ADAM10 downregulation is linked to hippocampal neuroinflammation and can exacerbate seizures [148].

### 4.5. Ketone Body Supplementation

No published clinical studies using ketone body supplementation to treat cognitive impairment with or without epilepsy are available to date. Preclinical studies in the *Kcna1*-null mouse model of temporal epilepsy with cognitive impairment demonstrated that the exogenous delivery of beta-hydroxy butyrate restored spatial memory performance in the Barnes Maze test and long-term potentiation in the hippocampus [6]. These effects were attributed to the improved health/function of brain mitochondria. Furthermore, ketone esters also improved working memory and re-learning in a mouse model of AD, as shown by the MWM test. By administering an open field test in addition to the MWM, Kashiwaya et al. [149] noted that these improvements in working memory, which is an aspect of executive functioning, and re-learning were independent of any influences that the ketone ester may have had on anxiety. The ketone ester also improved learning in a mouse model of Angelman’s syndrome [44]. 

### 4.6. Polyunsaturated Fatty Acid (PUFA) Supplementation

Even though preclinical studies and human epidemiological studies indicate that PUFAs have positive effects on cognition, clinical randomized controlled trials report conflicting results, complicating interpretations of their potential therapeutic usefulness. A recent meta-analysis of clinical trials in the elderly has provided a possible explanation for this frustrating situation. Overall, omega-3 PUFAs had no effect on global cognitive function; however, taking into account the starting baseline levels of omega-3 PUFAs revealed a clear association with improved cognitive function [150]. 

There are no clinical or preclinical studies of PUFAs’ effects on cognitive impairment in epilepsy. However, there are several preclinical studies in healthy and disease model animals. In healthy and aged rodents, diets supplemented with PUFAs improved their performance on several tests of memory, including spatial, object recognition, and associative memory [151,152,153]. DHA supplements seem to be more efficacious than EPA in improving memory and attenuating the proinflammatory cytokines associated with ageing [152]. Interestingly, PUFAs prevented sleep deprivation-induced cognitive impairment in otherwise healthy rats [154]. This was associated with the PUFA treatment’s prevention of a sleep deprivation-induced reduction in endogenous hippocampal antioxidants. Sleep disorders are often comorbid with epilepsy and sleep deprivation lowers seizure thresholds [155,156,157,158]; thus, PUFAs’ amelioration of cognitive impairments may be the result of disrupting the sleep disorder–cognitive impairment–epilepsy axis. 

### 4.7. Triheptanoin

Clinical or preclinical studies of triheptanoin’s effects on cognitive impairment with or without epilepsy are limited. One small uncontrolled clinical trial suggests that triheptanoin improves cognitive performance in Glucose Transporter Type I Deficiency [159]. Interestingly, preclinical evidence also suggests positive effects of triheptnoin on cognition as the addition of triheptanoin to a KD for 3 months enhanced object recognition in a V-maze by APP/PS1 mice, a model of AD [160].

## 5. Conclusions

Despite the overall scarcity of clinical data relating to the effect of the reviewed diets and supplements on neurological disorders comorbid with epilepsy, there is promising emerging evidence for their use in individual disorders. Research into the mechanisms of diverse neurological disorders indicate shared pathophysiologies that contribute to each disorder, including dysfunctional energy metabolism, neuroinflammation, oxidative stress, and gut dysbiosis. This may explain the effectiveness of the reviewed diets and supplements on what are thought to be vastly different disorders. The available clinical and preclinical data support the expectation that the use of dietary and select supplements will not only protect against seizures but also improve the comorbid symptoms of autism, affective disorders, and cognitive impairment. Indeed, it may be time to begin considering the presence of multiple disorders as a spectrum with one or more common final pathways that are mechanistically shared. From this perspective, utilizing dietary and metabolic approaches as adjuvants in concert with the current pharmacological armamentarium to treat epilepsy and its comorbidities is a logical strategy that is no longer an unconventional or radical idea. Further clinical and preclinical studies are warranted to test this hypothesis.

## Figures and Tables

**Table 1:** Summary of studies.

Therapy	Autism	Affective	Cognitive	Possible Mechanism of Action
Ketogenic Diet	P: + C: +	P: + C: +	P: +/− C: +/−	Brain glucose regulation Increased fatty acid metabolism Improved mitochondrial function Improved basal oxidative metabolism Induction of ketosis and ketone body generation Reduced neuroinflammatory cytokines Reduced oxidative stress and ROS generation
Medium-Chain Triglyceride	P: NA C: +	P: +/− C: NA	P: + C: +/−	Reduced oxidative stress Reduced serum cortisol
Low Glycemic Index	P: + C: NA	P: NA C: NA	P: NA C: NA	Reduction in markers of neuroinflammation and oxidative stress
Caloric Restriction	P: + C: NA	P: + C: +	P: + C: +/−	Increased antioxidant and anti-inflammatory mediators Increased mitochondria biogenesis Serotonin modulation Inhibition of mTOR signaling and downstream effects: Increased fatty acid utilization and ketone bodies Reduced oxidative stress and neuroinflammation Increased levels of metalloproteinase ADAM10, which regulates neuronal network homeostasis
**Supplementation Strategies**				
Ketone Bodies	P: + C: NA	P: + C: NA	P: + C: NA	Improved mitochondrial function Reduction in inflammatory cytokines and corticosterone
PUFAs	P: + C: +	P: + C: +	P: + C: +	Reduction in proinflammatory cytokines PPARgamma (peroxisome proliferator-activated receptor gamma) activation and downstream anti-inflammatory pathways Improved sleep
Triheptanoin	P: + C: NA	P: NA C: NA	P: + C: +	Improved basal oxidative metabolism Improved mitochondrial morphology Normalized serum leptin and insulin levels

P, preclinical animal studies; C, clinical studies; +, evidence of improvement; -, evidence of lack of effect; NA, no evidence available.
